# Personalised glucose therapy: glucose targets in critically ill patients with pre-existing poorly controlled type 2 diabetes

**DOI:** 10.1186/2197-425X-3-S1-A290

**Published:** 2015-10-01

**Authors:** P Kar, MP Plummer, R Bellomo, AJ Jenkins, AS Januszewski, K Lange, MJ Chapman, M Horowitz, AM Deane

**Affiliations:** Intensive Care Unit, Royal Adelaide Hospital, Adelaide, Australia; Discipline of Acute Care Medicine, University of Adelaide, Adelaide, Australia; Intensive Care Unit, The Austin Hospital, Melbourne, Australia; NHMRC Clinical Trials Centre, University of Sydney, Sydney, Australia; Discipline of Medicine, University of Adelaide, Adelaide, Australia; NHMRC Centre for Research Excellence, University of Adelaide, Adelaide, Australia

## Introduction

In patients without pre-existing diabetes, hyperglycaemia during critical illness is associated with adverse outcomes. However, recent observational data suggest that in patients with pre-existing poorly controlled type 2 diabetes (defined as an HbA1c ≥7%) prior to their acute illness, targeting glucose concentrations < 10mmol/l is associated with harm [1]. Accordingly a higher glucose target may benefit these patients.

## Objectives

To determine whether more liberal glucose targets in critically ill patients with pre-existing poorly controlled type 2 diabetes increases time weighted mean glucose concentration, attenuates hypoglycaemia, and appears overtly safe.

## Methods

Prospective, open-label, sequential period, pilot study of 86 patients with poorly controlled type 2 diabetes (admission HbA1c ≥ 7.0%) and a blood glucose concentration > 10mmol/L requiring admission to the Intensive Care Unit (ICU) and administration of insulin. The 'control' patients (n = 53) were consecutively admitted during a 6 month period and the 'intervention' patients (n = 33) were admitted during a subsequent 6 month period. During the 'control' period blood glucose was targeted between 6-10mmol/l, whereas during the 'intervention' period the target was 10-14mmol/l. Time weighted mean glucose was calculated and recorded blood glucose concentrations < 4.0mmol/l considered as an hypoglycaemic episode for each patient. Data are mean (SE) or median [IQR] and analysed using independent samples t-test, Mann-Whitney test, Chi-squared test, Linear and Logistic regression as appropriate.

## Results

The groups were well matched in terms of age (Control: 64.0 (2.0) vs Intervention: 62.6 (2.0) years), admission HbA1c (8.5 (0.2) vs 8.8 (0.2)%), APACHE II score (20.4 (1.0) vs 20.5 (1.2)) and Charlson Comorbidity Index (4.5 (0.3) vs 4.3 (0.3)). More liberal targets resulted in greater time weighted mean glucose concentrations (TWGlucose_day0-7_ 9.3 (0.3) vs 10.2 (0.4) mmol/l, P = 0.04) and there was a trend towards fewer patients with hypoglycaemic episodes (34% vs 16% of patients, P = 0.07). There was no difference in ICU mortality (10 [19%] vs 6 [18%], P = 0.94) or 90-day mortality (19 [36%] vs 12 [36%], P = 0.96). ICU length of stay was shorter in the 'control' period (3.5 [4.0] vs 6.3 [8.7] days, P = 0.01) with significance persisting when adjusting for APACHE scores and Charlson Comorbidity Index (P = 0.04).

**Figure 1:**
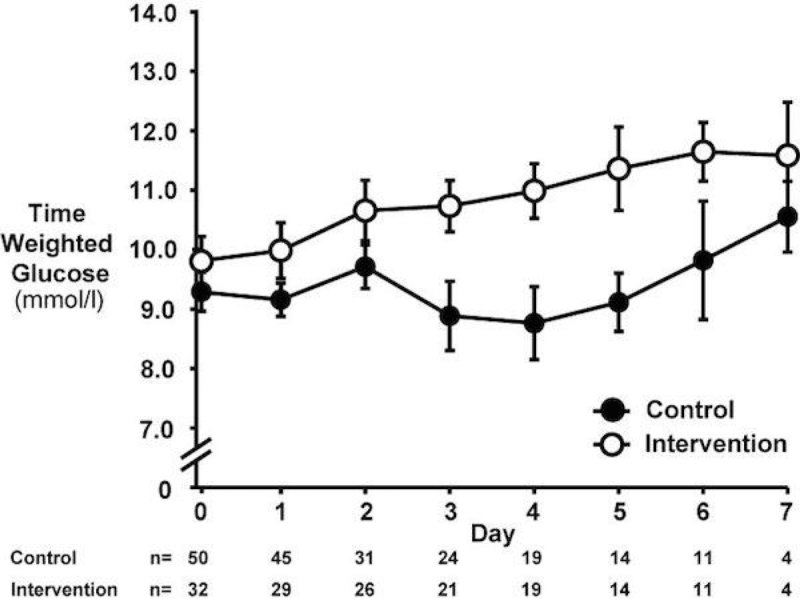
**Mean time weighted glucose between groups.**

## Conclusions

In critically ill patients with pre-existing poorly controlled type 2 diabetes, more liberal glucose targets increase mean glucose concentrations may reduce the incidence of insulin- induced hypoglycaemia, and appear to be overtly safe. Prospective studies using larger cohorts are indicated.

## Grant Acknowledgment

Dr Kar is supported by a Royal Adelaide Hospital A.R. Clarkson Scholarship
